# Presynaptic density determined by SV2A PET is closely associated with postsynaptic metabotropic glutamate receptor 5 availability and independent of amyloid pathology in early cognitive impairment

**DOI:** 10.1002/alz.13817

**Published:** 2024-04-18

**Authors:** Jie Wang, Qi Huang, Kun He, Junpeng Li, Tengfei Guo, Yang Yang, Zengping Lin, Songye Li, Greet Vanderlinden, Yiyun Huang, Koen Van Laere, Yihui Guan, Qihao Guo, Ruiqing Ni, Binying Li, Fang Xie

**Affiliations:** ^1^ Department of Nuclear Medicine & PET Center Huashan Hospital, Fudan University Shanghai China; ^2^ Institute of Biomedical Engineering, Shenzhen Bay Laboratory Shenzhen China; ^3^ Beijing United Imaging Research Institute of Intelligent Imaging Beijing China; ^4^ Central Research Institute, United Imaging Healthcare Group Co., Ltd Shanghai China; ^5^ Department of Radiology and Biomedical Imaging PET Center Yale University School of Medicine New Haven Connecticut USA; ^6^ Department of Imaging and Pathology Nuclear Medicine and Molecular Imaging, KU Leuven Leuven Belgium; ^7^ Department of Gerontology Shanghai Jiao Tong University Affiliated Sixth People's Hospital Shanghai China; ^8^ Institute for Biomedical Engineering, University of Zurich & ETH Zurich Zurich Switzerland; ^9^ Institute for Regenerative Medicine University of Zurich Zurich Switzerland; ^10^ Department of Neurology and Institute of Neurology Ruijin Hospital, Shanghai Jiao Tong University School of Medicine Shanghai China; ^11^ State Key Laboratory of Medical Neurobiology and MOE Frontiers Center for Brain Science, Fudan University Shanghai China

**Keywords:** Alzheimer's disease, amyloid deposition, cognition, mGluR5 availability, PET/MR, synaptic density

## Abstract

**INTRODUCTION:**

Metabotropic glutamate receptor 5 (mGluR5) is involved in regulating integrative brain function and synaptic transmission. Aberrant mGluR5 signaling and relevant synaptic failure play a key role in the pathophysiological mechanism of Alzheimer's disease (AD).

**METHODS:**

Ten cognitively impaired (CI) individuals and 10 healthy controls (HCs) underwent [^18^F]SynVesT‐1 and [^18^F]PSS232 positron emission tomography (PET)/magnetic resonance to assess synaptic density and mGluR5 availability. The associations between mGluR5 availability and synaptic density were examined. A mediation analysis was performed to investigate the possible mediating effects of mGluR5 availability and synaptic loss on the relationship between amyloid deposition and cognition.

**RESULTS:**

CI patients exhibited lower mGluR5 availability and synaptic density in the medial temporal lobe than HCs. Regional synaptic density was closely associated with regional mGluR5 availability. mGluR5 availability and synaptic loss partially mediated the relationship between amyloid deposition and cognition.

**CONCLUSIONS:**

Reductions in mGluR5 availability and synaptic density exhibit similar spatial patterns in AD and are closely linked.

**Highlights:**

- Cognitively impaired patients exhibited lower mGluR5 availability and synaptic density in the medial temporal lobe than HCs.
- Reductions in mGluR5 availability and synaptic density exhibit similar spatial patterns in AD.
- Regional synaptic density was closely associated with regional mGluR5 availability.
- mGluR5 availability and synaptic loss partially mediated the relationship between amyloid deposition and global cognition.
- With further research, modulating mGluR5 availability might be a potential therapeutic strategy for improving synaptic function in AD.

## 1 BACKGROUND

Alzheimer's disease (AD) is an age‐related, progressive neurodegenerative disorder characterized by memory loss and cognitive decline. Despite its alarming and increasing prevalence, treatments that either modify or reverse AD's progression are limited.^1^ The neuropathological hallmarks of AD are the presence of extracellular senile amyloid beta (Aβ) plaques and intracellular neurofibrillary tangles composed of hyperphosphorylated tau.^2^ Accumulating evidence suggests that synaptic dysfunction and loss are also major pathological mechanisms of mild cognitive impairment (MCI) in early AD and represent a structural basis for AD‐related dementia.^3^, ^4^ Synaptic loss has been identified as a major pathological correlate of cognitive impairment in AD.^5^

Synaptic vesicle glycoprotein 2A (SV2A) is an integral 12‐transmembrane‐domain glycoprotein expressed in synaptic vesicles throughout the brain and could serve as a suitable biomarker for measuring the synaptic density.^6^, ^7^ Quantification of synaptic density in vivo is possible by positron emission tomography (PET) with specific SV2A radioligands. The synaptic [^11^C]UCB‐J PET radiotracer has a nanomolar affinity for SV2A, allowing direct measurement of synaptic density in vivo.^8^, ^9^ [^18^F]SynVesT‐1 is a novel [^18^F] radiolabeled difluoro‐analog of [^11^C]UCB‐J with similar affinity and kinetics.^10^ Previous studies found widespread reductions in SV2A binding in the medial temporal lobe and neocortical brain regions in early AD patients compared to healthy controls (HCs). Patients with amnestic MCI due to AD (aMCI) show decreased [^11^C]UCB‐J binding mainly in the medial temporal lobe.^11^, ^12^, ^13^, ^14^

Metabotropic glutamate receptor 5 (mGluR5) is an excitatory G‐protein coupled receptor expressed primarily in postsynaptic nerve terminals,^15^, ^16^, ^17^ as well as in non‐neuronal cells (such as microglia and astrocytes), that modulates neuronal excitation, synaptic transmission, and neuronal development.^7^ Evidence from preclinical studies of AD patients indicates that aberrant mGluR5 signaling is associated with Aβ oligomer‐induced early synaptic failure.^18^ Renner et al. reported that Aβ oligomer‐induced abnormal mGluR5 production and overstabilization elevated intracellular calcium levels, consequently leading to synapse deterioration.^19^ Recognition of mGluR5 as a mediator of AD pathology as a potentially important therapeutic target^20^ has led to the application of PET to investigate the changes in mGluR5 expression in patients with AD. Significant reductions in mGluR5 levels in the hippocampus have been found in aMCI and early AD, as measured by different mGluR5 radiotracers, including [^18^F]FPEB and [^11^C]ABP688.^21^, ^22^

Both presynaptic SV2A and postsynaptic mGluR5 receptors play key roles in AD and may interact synergistically.^23^ Previous results revealed that mGluR5 activity was reduced in AD mice and that selective modulation of mGluR5 reversed synaptic loss,^24^ indicating that synaptic loss was mGluR5 dependent. To date, the relationship between mGluR5 and synaptic loss as well as their interactions with Aβ deposition in vivo in AD patients remain unknown.

In this study, we evaluated decreases in synaptic density using [^18^F]SynVesT‐1 and mGluR5 availability using [^18^F]PSS232, a previously reported selective radioligand for the measurement of mGluR5 expression in human brains.^25^ We hypothesized that in cognitively impaired (CI) individuals, cerebral mGluR5 availability and synaptic density would be decreased, especially in the medial temporal lobe, and that the changes would have a similar spatial pattern as but are independent of amyloid pathology distribution. Furthermore, we hypothesized that both mGluR5 availability and synaptic loss would correlate with the degree of Aβ deposition, hippocampal atrophy, and cognitive dysfunction.

## 2 MATERIALS AND METHODS

### 2.1 Participants

Twenty participants, including 10 CI individuals and 10 HCs, were recruited from the memory clinic of Shanghai Jiao Tong University Affiliated Ruijin Hospital and communities in Shanghai from September 2021 to October 2023. They volunteered to join the study cohort through an advertisement on a public board or were recruited when they or their family members reported a decline in their memory. All participants underwent [^18^F]florbetapir PET/computed tomography (CT), [^18^F]SynVesT‐1 and [^18^F]PSS232 PET/magnetic resonance imaging (MRI) scans, and neuropsychological assessments. All examinations were completed within a month. This study was approved by the Institutional Ethics Review Board of Huashan Hospital, Fudan University, and Shanghai Jiao Tong University Affiliated Ruijin Hospital. Written informed consent was obtained from the participants (or their caretakers in case of inability to provide consent) before inclusion.

The inclusion criteria for all participants were as follows: (1) age range 60 to 80 years, regardless of gender or years of education; (2) patients matched with one caregiver and willing to complete questionnaire assessment; (3) AD diagnosis according to 2018 National Institute on Aging and Alzheimer's Association (NIA‐AA) diagnostic criteria^26^; MCI was diagnosed according to Jak and Bondi's revised criteria because their cognitive impairment is not severe enough to meet the criteria for dementia due to AD. Briefly, a diagnosis of MCI was given if the participant met one of the following criteria: (1) impairment (defined as >1 standard deviation [SD] below the age‐corrected normative mean) on two of the six neuropsychological tests in the same cognitive domain; and (2) impairment (>1 SD) in each of the three cognitive domains^27^, ^28^, ^29^; individuals who had no impairment in any cognitive domains were considered HCs^29^, ^30^; (3) all subjects first completed [18F]florbetapir PET/CT scan, and only CI individuals with positive Aβ PET and HC with negative Aβ PET underwent subsequent PET/MRI scans; (5) only patients with mild AD were included in study, according to global Clinical Dementia Rating (CDR), with CDR = 1.^31^

**RESEARCH IN CONTEXT**

1. **Systematic review**: Metabotropic glutamate receptor 5 (mGluR5) is involved in regulating the integrative brain function and synaptic transmission. Aberrant mGluR5 signaling and relevant synaptic failure play a key role in the initial pathophysiological mechanism of Alzheimer's disease (AD). We searched PubMed, but we found no literature reporting the association of mGluR5 availability with synaptic density.
2. **Interpretation**: In this study, we found that cognitively impaired patients exhibited lower mGluR5 availability and synaptic density in the medial temporal lobe. Regional synaptic density was closely associated with regional mGluR5 availability. mGluR5 availability and synaptic loss partially mediated the relationship between amyloid deposition and global cognition.
3. **Future directions**: The future research direction is to observe the longitudinal changes of mGluR5 availability and synaptic density and investigate how tau tangles affect the mGluR5 expression and synaptic loss to further understand the mGluR5 expression and synaptic loss during AD progression and its underlying mechanisms.

### 2.2 Exclusion criteria

The exclusion criteria for all participants were as follows: (1) a current major psychiatric diagnosis such as severe depression or anxiety; (2) a history of other neurological conditions that could cause cognitive decline (eg, cerebrovascular disease, brain tumors, Parkinson's disease, or epilepsy) other than AD spectrum disorders; (3) a history of other diseases that could cause cognitive decline (eg, thyroid dysfunction, severe anemia, syphilis, or human immunodeficiency virus); (4) a history of psychosis or inherited intellectual disability; (5) a history of cognitive decline caused by traumatic brain injury; and (6) a history of conditions that would prevent completion of the study protocol or presented contraindications for MRI.^32^ Furthermore, participants with a history of smoking were excluded.^33^

### 2.3 Neuropsychological assessment

All participants underwent a comprehensive neuropsychological assessment revised for use in the Chinese population.^34^, ^35^ The Mini‐Mental State Examination (MMSE) was used to assess global cognition, six neuropsychological tests in three cognitive domains were administered, and a global CDR was used (CDR = 0.5 for MCI, >0.5 for AD diagnosis). The auditory verbal learning test (AVLT), 30‐min long delayed free recall of the AVLT (AVLT‐LDR, 12 items), and AVLT‐recognition (24 items) were performed to test memory; the animal fluency test (AFT, total score) and Boston Naming Test (BNT, 30 items) were used to evaluate language; and parts A and B of the shape trials test (STT) (time to completion) were performed to assess executive function.^36^, ^37^

### 2.4 PET and MR neuroimaging

[^18^F]SynVesT‐1, [^18^F]PSS232, and [^18^F]florbetapir were produced by the Department of Nuclear Medicine & PET Center, Huashan Hospital, Fudan University, according to the manufacturer's protocol and under good manufacturing practice conditions. [^18^F]SynVesT‐1 and [^18^F]PSS232 PET/MR scans were acquired on a 3T PET/MR machine (uPMR790, United Imaging Healthcare, Shanghai, China). A 30‐min static PET scan was started 60 min following injection of [^18^F]SynVesT‐1 (185 ± 17.8 MBq) and 30 min following injection of [^18^F]PSS232(185 ± 15.6 MBq). The static PET data were reconstructed using all list mode events with the following parameters: field of view (FOV) = 300 × 300 mm^2^, matrix size = 256 × 256, voxel size = 2.0 × 2.0 × 2.0 mm^3^, and the ordered subset‐expectation maximization algorithm (20 subsets and four iterations). Data were reconstructed after correction for random, dead time, scatter, and attenuation. For MRI, a 3D Dixon sequence was acquired for attenuation correction, and a T1‐weighted MR scan was simultaneously acquired using the following parameters: repetition time = 7200 ms, echo time = 3.0 ms, flip angle = 10°, acquisition matrix = 256 × 329, in‐plane resolution = 1 × 1 mm, slice thickness = 1 mm, and slices = 176.^38^

[^18^F]florbetapir PET/CT was conducted (Biograph Myth Flow, Siemens, Erlangen, Germany) with previously described parameters.^39^ Subjects were intravenously injected with 370 ± 37 MBq of [^18^F]florbetapir, and 50 min after the injection, a 20‐min PET acquisition scan with a low‐dose CT scan for attenuation correction was performed. After the acquisition, all PET images were reconstructed utilizing a filtered back‐projection algorithm with the following parameters: image size 256 × 256, zoom factor 2.00, Gaussian filter, full width at half maximum (FWHM) of 3.5 mm, and corrections for decay, normalization, dead time, attenuation, scatter, and random coincidences.^40^

### 2.5 Data preprocessing

We used SPM12 (Wellcome Centre for Neuroimaging, London, UK; https://www.fil.ion.ucl.ac.uk/spm) for PET image preprocessing following a previously described procedure.^40^ After the reorientation of PET and T1‐weighted MR images, the PET images were co‐registered to the individual T1‐weighted images. Then the T1‐weighted images were warped into the standard Montreal Neurological Institute (MNI) space and segmented into gray matter (GM), white matter (WM), and cerebrospinal fluid (CSF); the labeled images were used for partial volume correction (PVC) of all three types of PET images using the Muller‐Gartner method,^41^ and the resulting normalization parameters were applied to the corresponding PET images. The normalized PET images were smoothed by a Gaussian filter with an FWHM equal to 8 mm.^39^ All three types of PET images, with PCV and without PVC, were included in the analysis; one analysis used only PET images without PVC, and another analysis used all images with PVC.

All PET imaging variables were computed in the standard space. PET data were quantified by determining the standardized uptake value ratio (SUVr) using the whole cerebellum as the reference region for [^18^F]SynVesT‐1 and [^18^F]PSS232 and cerebellar GM as the reference region for [^18^F]florbetapir. Volumes of interest (VOIs) were defined by an automated anatomical labeling (AAL) atlas and applied to the PET data, including the frontal, lateral parietal, lateral temporal, medial temporal, and occipital lobes, and precuneus and posterior cingulate cortex; the composite cortical VOI was calculated;  the sum of the aforementioned VOIs as the global cortex VOI.^42^ The SUVr of Aβ deposits in the composite cortical VOI was calculated as the proportion of the mean signal intensity of the whole GM to that of the cerebellar GM. The global [^18^F]PSS232 and [^18^F]SynVesT‐1 SUVr values were calculated as the proportion of the mean activity concentration of all of the aforementioned regions to that of the cerebellum.^43^ Three board‐certified nuclear medicine physicians, including two with mid‐level titles and one with a senior‐level title, visually interpreted all [^18^F]florbetapir images in a binary (positive/negative) way according to visual rating guidelines for Aβ PET interpretation, and they were all blinded to clinical, demographic, and neurological information. The results were considered positive if at least two out of three physicians made the same assessment.^32^ Hippocampal volume was calculated by FreeSurfer version 4.3 (https://surfer.nmr.mgh.harvard.edu)^44^ and normalized to the intracranial volume to control for cerebral size differences. The calculated hippocampal volume ratio (HPVR) was used for further analysis.

### 2.6 *Post mortem* human brain tissue

*Post mortem* analyses were performed on brain tissues from independent samples of five AD patients, with a clinical diagnosis confirmed by pathological examination, and five non‐demented controls (detailed information in Table S1). Paraffin‐embedded hippocampal tissue blocks were obtained from the Netherlands Brain Bank (NBB), Netherlands. All materials had been collected from donors or from patients from whom written informed consent for a brain autopsy and the use of the materials and clinical information for research purposes had been obtained by the NBB. The study was conducted according to the principles of the Declaration of Helsinki and subsequent revisions. All experiments on autopsied human brain tissue from the NBB were carried out with permission from the regional human ethics committee and the medical ethics committee of the VU Medical Center.

### 2.7 Multiplex immunofluorescence staining and microscopy

Paraffin‐embedded human brain sections (4 μm) were deparaffinized in xylene and rehydrated before antigen retrieval (in ethylenediaminetetraacetic acid buffer) in a microwave for 15 min. Multiplex immunofluorescence staining was performed by Alpha Painter X30. The primary and secondary antibodies used are listed in Table S2 and included rabbit anti‐mGluR5 (1:500), mouse anti‐Aβ1‐16, 6E10 (1:1000), and rabbit anti‐SV2A (1:500). The sections were incubated with primary antibodies for 1 h at 37°C. The AlphaTSA Multiplex IHC Kit was used for visualization. After each staining cycle, heat‐induced epitope retrieval was performed to remove all the antibodies, including primary and secondary antibodies. The nuclei were counterstained with 4′,6‐diamidino‐2‐phenylindole (DAPI) for 5 min and mounted with a mounting medium. Immunohistochemical staining using 4G8, anti‐Aβ17‐24 (1:4000) was performed on the adjacent hippocampal sections. The immunohistochemistry‐ and immunofluorescence‐stained brain sections were imaged at ×20 magnification with a Zeiss Axioscan 7 slide scanner (Zeiss, Germany) with the same acquisition settings. Images were analyzed using Qupath and ImageJ (National Institutes of Health). The average fluorescence intensity was quantified using HALO (version 3.5; Indica Labs).

### 2.8 Statistical analysis

Voxelwise group comparisons were conducted in SPM12, and analyses of clinical and VOI data were conducted using SPSS (version 23.0, IBM, Armonk, NY, USA). All of the continuous data were normally or approximately normally distributed according to the Kolmogorov‒Smirnov test, with *p* values >0.05. Differences in demographic data, neuropsychological scores, and SUVr values were analyzed using Student's *t* test. The differences in fluorescence intensity of Aβ (6E10), mGluR5, and SV2A staining in the hippocampus slices were analyzed using a Mann‐Whitney U test. Correlations of global Aβ deposition, HPVRs, and MMSE scores with mGluR5 availability and synaptic density were analyzed by a multivariate linear regression (MLR) model based on voxelwise analysis and Pearson correlation analysis of VOI SUVr data. Correlations of mGluR5 availability with synaptic density were analyzed by MLR based on voxelwise analysis and Pearson correlation analysis of SUVr data for both regional and composite cortical VOIs. To determine the impact of mGluR5 availability and synaptic density on the relationship between Aβ and global cognition, we conducted mediation analyses to test whether the relationship between Aβ‐PET data and MMSE scores could be explained by the mGluR5 PET and SV2A PET data using PROCESS version 4.1. The significance of this mediating effect was assessed by calculating bias‐corrected 95% confidence intervals (CIs) using bootstrapping (5000 resamples). VOI‐based statistical analysis was performed both with and without PVC of PET images. For voxelwise analyses, the statistical level was set at *p* < 0.001, and the minimum cluster extent ke was ≥100 voxels without correction for multiple comparisons. Because this was an exploratory study with a small sample size, for VOI analyses, *p* < 0.05 was considered to indicate a significant difference (two‐sided) without correction for multiple comparisons.

## 3 RESULTS

### 3.1 Demographic characteristics and clinical assessments

A total of 20 participants, including 10 CI individuals (five with mild AD and five with MCI) and 10 HCs, were included in our study. The CI individuals had lower MMSE scores (22.7 ± 2.2 vs 28.9 ± 1.2, *p *< .001), AVLT‐LDR scores (0.7 ± 1.5 vs 5.5 ± 3.1, *p* = 0.001), AVLT‐recognition scores (15.2 ± 4.2 vs 21.6 ± 2.3, *p* < 0.001), and AFT scores (6.5 ± 2.8 vs 9.8 ± 3.9, *p* = 0.043) than the HCs (Table 1). There were no significant differences in female proportion, age, years of education, or other comprehensive neuropsychological scores between the CI individuals and HCs.

**TABLE 1:** Demographic information and clinical characteristics.

Characteristic	CI	HCs	*P* value
Number	10	10	
Female	80%(8/10)	70%(7/10)	0.693
Age (years)	70.6 ± 6.4	67.7 ± 6.9	0.344
Education (years)	10.9 ± 3.3	11.8 ± 2.5	0.501
MMSE score	22.7 ± 2.2	28.9 ± 1.2	**<0.001**
AVLT‐LDR score	0.7 ± 1.5	5.5 ± 3.1	**0.001**
AVLT‐recognition score	15.2 ± 4.2	21.6 ± 2.3	**<0.001**
BNT score	19.4 ± 6.4	23.7 ± 4.2	0.093
AFT score	6.5 ± 2.8	9.8 ± 3.9	**0.043**
STT‐A score	192.6 ± 295	60.6 ± 19	0.176
STT‐B score	416 ± 422	150 ± 56	0.054
[^18^F]florbetapir SUVr of global cortex	1.57 ± 0.16	1.25 ± 0.06	**<0.001**
No. amyloid‐positive/amyloid‐negative scans (visual)	10/0	0/10	**<0.001**
HPVR	0.0043 ± 0.0009	0.0053 ± 0.0008	**0.016**

*Note*: Bolded *p* values indicate statistical significance (*p* < 0.05).

Abbreviations: AFT, animal fluency test; ALVT, auditory verbal learning test; BNT, Boston Naming Test; CI, cognitively impaired individuals; HPVR, hippocampal volume ratio; STT, shape trials test; SUVr, standardized uptake value ratio.

### 3.2 Group differences in mGluR5 availability, synaptic density, amyloid deposition, and hippocampal volume

**FIGURE 1:**
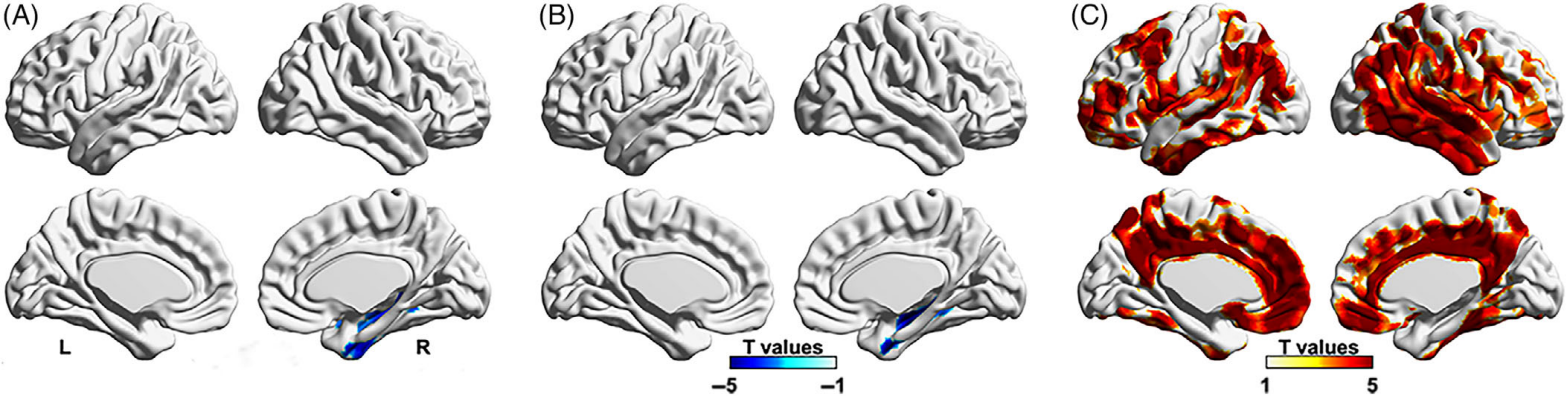
Voxelwise‐based differences in (A) mGluR5 availability, (B) synaptic density, and (C) amyloid deposition between CI individuals and HCs. The colors indicate the T value of the differences between groups with a statistical threshold of *p* < 0.001 and a minimum cluster extent ke of ≥100 voxels according to voxelwise analysis. The analyses were adjusted for age, gender, and years of education as covariates.

As shown in Figure 1, according to a voxelwise‐based analysis, we found that the CI individuals had lower mGluR5 availability and synaptic density in the medial temporal lobe than the HCs. Similar results were obtained by VOI analysis; the CI individuals had a lower [^18^F]PSS232 SUVr (2.67 ± 0.25 vs 3.07 ± 0.15, *p* = 0.001) and [^18^F]SynVesT‐1 SUVr (0.92 ± 0.05 vs 1.02 ± 0.06, *p* = 0.004) than the HCs. We obtained similar results after PVC of PET images (Figure S1). The CI individuals had higher global Aβ deposition than the HCs according to voxelwise analysis and VOI‐based analysis (1.57 ± 0.16 vs 1.25 ± 0.06, *p* < 0.001). A smaller HPVR was also observed in the CI individuals (0.0043 ± 0.0009 vs 0.0053 ± 0.0008, *p* = 0.016) than in the HCs.

### 3.3 Reduced mGluR5 and SV2A levels in hippocampal tissues from AD patients compared to HCs

**FIGURE 2:**
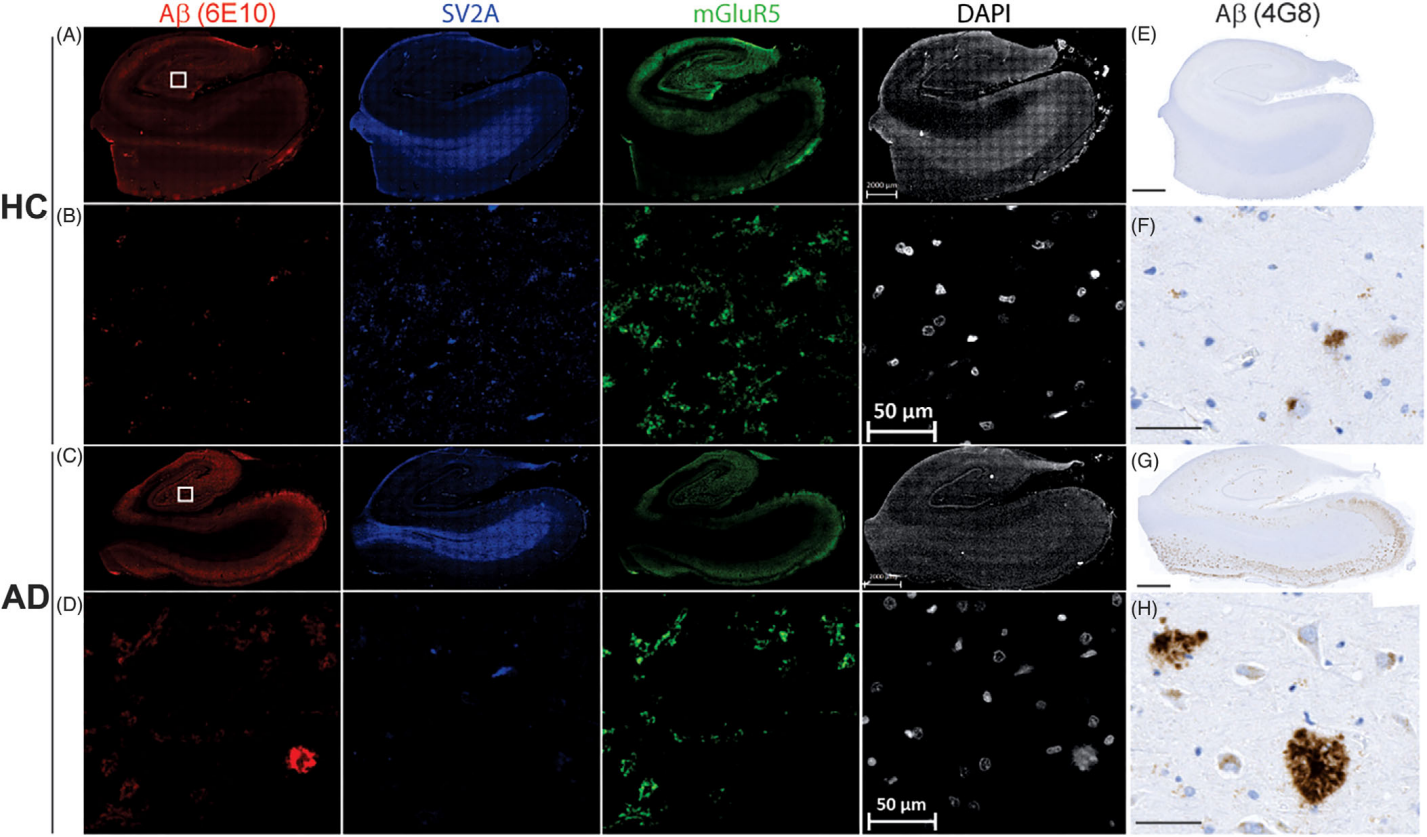
Reduced levels of mGluR5 and SV2A expression in hippocampus of AD patients in presence of amyloid plaques compared to that of HCs. (A–D) Presence of diffuse and dense Aβ plaques (6E10 staining, red), with relatively lower fluorescence intensities of SV2A (blue) and mGluR5 (green) in hippocampus of AD patients than that of HCs. (E–H) Representative 4G8 immunochemical staining for Aβ on hippocampus sections from HC and AD patients. (B, D, F, H) magnified images of cornu ammonis 4 (CA4). The nucleus was counterstained with DAPI (white). Scale bar = 2000 µm (A, C, E, G), and 50 µm (B, D, F, H).

The expression of mGluR5 and synaptic density (SV2A) were evaluated in *post mortem* hippocampal slices from AD patients and HCs using multiplex immunofluorescence staining (Figure 2). 6E10‐ and 4G8‐positive Aβ plaques (both diffuse and dense) were observed in the hippocampus of AD patients but seldom found in that of HCs. Expression of mGluR5 and SV2A was observed throughout the hippocampus but was lower in AD patients than in HCs. We further analyzed the fluorescence intensity of Aβ, mGluR5, and SV2A staining in the hippocampus slices. The average fluorescence intensity for 6E10‐positive Aβ was higher in the hippocampus of AD patients than HCs (148.65 ± 29.34 vs 38.67 ± 18.24, *p* < 0.001), while the average fluorescence intensity for mGluR5 and SV2A was lower in the hippocampus of AD patients than HCs (51.86 ± 25.10 vs 116.21 ± 38.21, *p* = 0.016; 47.73 ± 18.90 vs 86.65 ± 15.16, *p* = 0.008) (Figure S2).

### 3.4 Associations of mGluR5 availability with synaptic density in CI individuals and HCs

**FIGURE 3:**
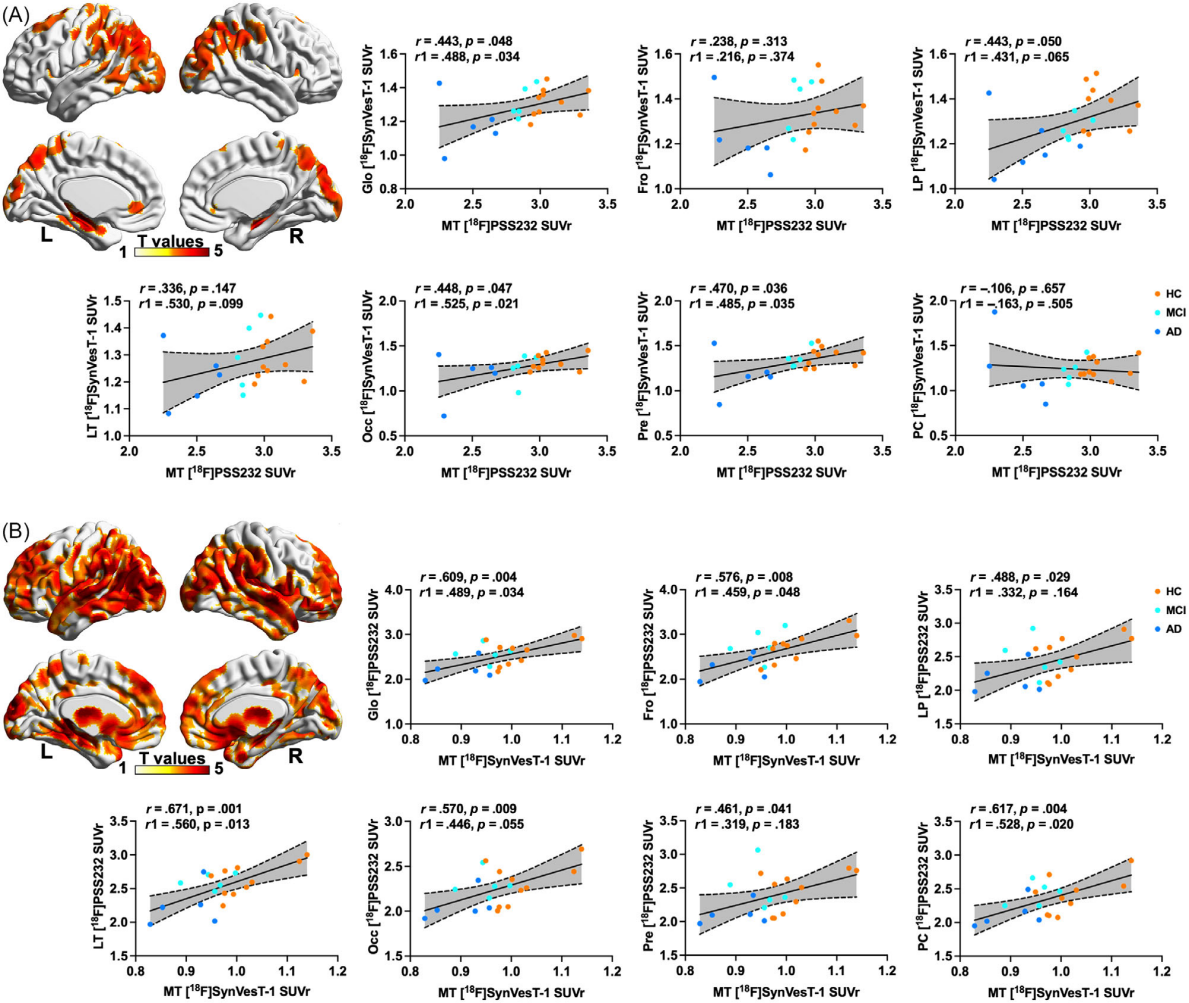
Associations between mGluR5 availability and synaptic density in whole cohort. (A) Associations of mGluR5 availability in medial temporal lobe with synaptic density. (B) Associations of synaptic density in medial temporal lobe and mGluR5 availability. Colors represent T value with statistical threshold of *p* < 0.001 and minimum cluster extent ke of ≥100 voxels. Dashed lines represent 95% confidence intervals of best‐fit lines. r: with age, education years, and gender as covariates; r1: with addition of global amyloid deposition as covariate. GC: global cortex; LP: lateral parietal lobe; LT: lateral temporal lobe; MT: medial temporal lobe; PC: posterior cingulate; Fro: frontal lobe; Occ: Occipital lobe; Pre: Precuneus; SUVr: standardized uptake value ratio.

mGluR5 availability in the medial temporal lobe of CI individuals and HCs was positively associated with synaptic density in the entire cortex (r = 0.443, *p* = 0.048), lateral parietal lobe (r = 0.443, *p* = 0.050), occipital lobe (r = 0.448, *p* = 0.047), and precuneus (r = 0.470, *p* = 0.036). The associations persisted when the global cortex VOI SUVr was used as a covariate (Figure 3A). The result was similar to what was revealed by voxelwise analysis.

We also found that synaptic density in the medial temporal lobe of CI individuals and HCs was positively associated with global cortical mGluR5 availability (r = 0.609, *p* = 0.004) and regional mGluR5 availability in the frontal (r = 0.576, *p* = 0.008), lateral parietal (r = 0.488, *p* = 0.029), lateral temporal (r = 0.671, *p* = 0.001), and occipital lobes (r = 0.570, *p* = 0.009) and precuneus (r = 0.461, *p* = 0.041) and posterior cingulate cortex (r = 0.617, *p* = 0.004), and these associations were independent of global amyloid deposition. A similar result was obtained by voxelwise analysis (Figure 3B). Similar results were obtained after PVC of the PET images (Table S3 and Figure S3).

Global mGluR5 availability in CI individuals and HCs was positively associated with synaptic density in the composite cortical VOI (r = 0.587, *p* = 0.007), frontal (r = 0.565, *p *= .009), lateral parietal (r = 0.539, *p* = 0.014), and medial temporal lobes (r = 0.609, *p* = 0.004) and precuneus (r = 0.515, *p* = 0.020) (Figure S4A). The synaptic density in the composite cortical VOI was positively associated with regional mGluR5 availability in the frontal (r = 0.607, *p* = 0.005), lateral temporal (r = 0.535, *p* = 0.015), medial temporal (r = 0.443, *p* = 0.048), and occipital lobes (r = 0.520, *p* = 0.019) and p posterior cingulate crus (r = 0.447, *p* = 0.033), and these associations were independent of global amyloid deposition. A similar result was obtained by voxelwise analysis (Figure S4B) and after applying PVC to the PET images (Table S3 and Figure S3).

**FIGURE 4:**
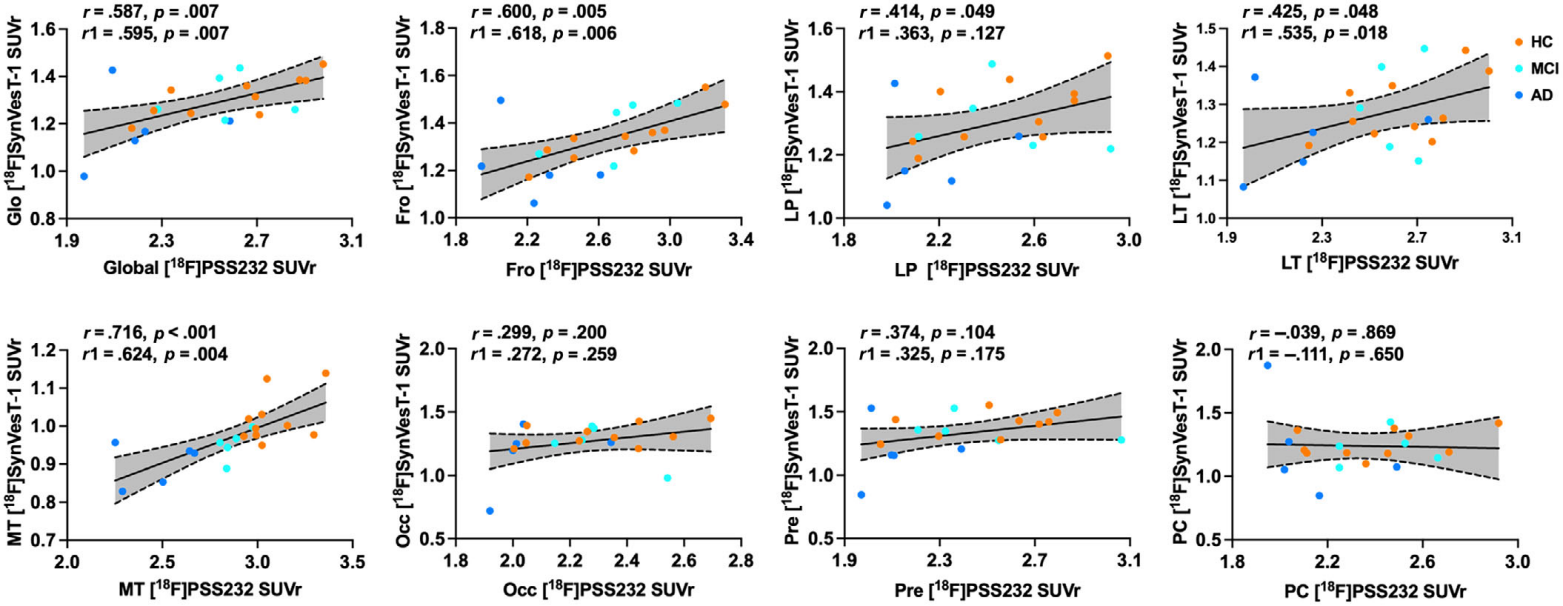
Associations between regional mGluR5 availability and regional synaptic density. Pearson's correlation analysis was performed; dashed lines represent 95% confidence intervals of best‐fit lines. r: with age, education years, and gender as covariates; r_1_: with addition of global amyloid deposition as covariate. GC: global cortex; LP: lateral parietal lobe; LT: lateral temporal lobe; MT: medial temporal lobe; PC: posterior cingulate; Fro: frontal lobe; Occ: Occipital lobe; Pre: Precuneus; SUVr: standardized uptake value ratio.

Regional mGluR5 availability in CI individuals and HCs was positively associated with regional synaptic density in the frontal (r = 0.600, *p* = 0.005), lateral parietal (r = 0.414, *p* = 0.049), medial temporal (r = 0.716, *p* < 0.001), and lateral temporal lobes (r = 0.425, *p* = 0.048). A similar result was obtained by analysis after applying PVC to PET images (Figure 4 and Figure S5).

### 3.5 Associations of global amyloid deposition with mGluR5 availability and synaptic density

**FIGURE 5:**
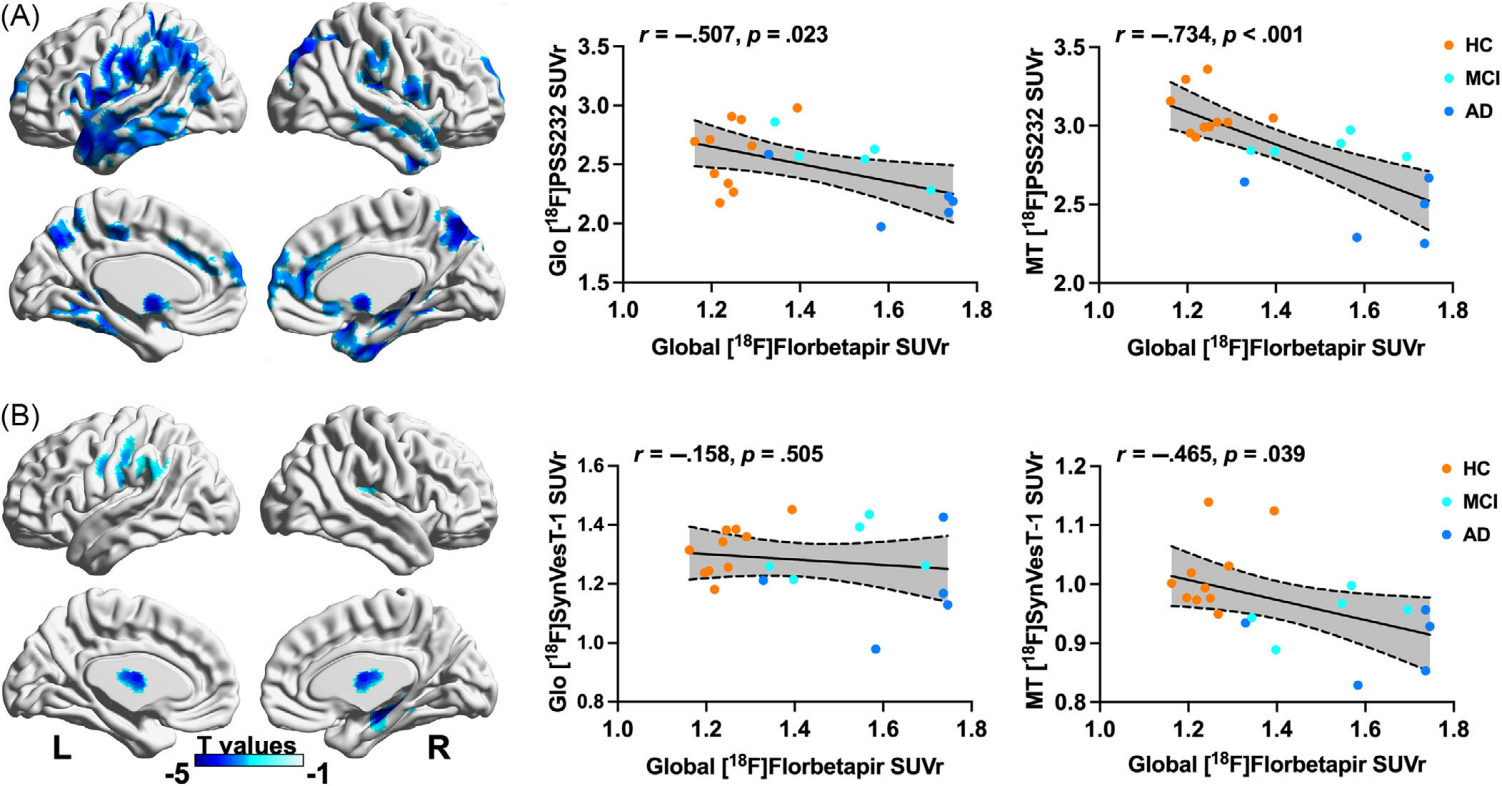
Associations of global amyloid deposition with (A) mGluR5 availability and (B) synaptic density in whole cohort. (A) Associations of global amyloid with mGluR5 availability. (B) Associations of global amyloid with synaptic density. Colors represent T value with statistical threshold of *p* < 0.001 and minimum cluster extent ke of ≥100 voxels. Dashed lines represent 95% confidence intervals of best‐fit lines. Analyses were adjusted for age, gender, and years of education as covariates. Glo: global cortex; MT: medial temporal lobe; PC: posterior cingulate.

Global cortical amyloid deposition was negatively associated with mGluR5 availability mainly in the lateral parietal and temporal lobes according to voxelwise analysis. Based on VOI analysis, we found that global amyloid deposition was negatively associated with mGluR5 availability in the entire cortex (r = −0.507, *p* = 0.023) and medial temporal lobe (r = −0.734, *p* < 0.001; Figure 5A). When the [^18^F]SynVesT‐1 SUVr of the entire cortex was added as a covariate, these associations still existed (r = −0.518, *p* = 0.022; r = −0.750, *p* < 0.001); when the [^18^F]SynVesT‐1 SUVr of the medial temporal lobe was added as a covariate, global amyloid deposition was only negatively associated with mGluR5 availability in the medial temporal lobe (r = −0.648, *p* = 0.003).

Global cortical amyloid deposition was negatively associated only with synaptic density in the right medial temporal lobe, left lateral parietal lobe, and bilateral insula according to voxelwise analysis (Figure 5B). In contrast, global cortical amyloid deposition was negatively associated with synaptic density in the medial temporal lobe (r = −0.465, *p* = 0.039) but not in the entire cortex (r = −0.158, *p* = 0.505) according to VOI‐based analysis. When the [^18^F]PSS232 SUVr of the entire cortex or medial temporal lobe was added as a covariate, there was no association between global cortical amyloid deposition and synaptic density. Similar results were obtained after PVC of the PET images (Table S3 and Figure S3).

### 3.6 Associations of HPVR and cognition with mGluR5 availability and synaptic density

The HPVR was positively associated with mGluR5 availability, mainly in the bilateral temporal lobes and insula according to voxelwise analysis. Based on VOI analysis, the HPVR was positively associated with regional mGluR5 availability in the lateral temporal (r = 0.558, *p* = 0.009) and medial temporal lobes (r = 0.656, *p* = 0.002) (Figure S6A and S7A). The HPVR was positively associated with synaptic density mainly in the lateral parietal and temporal lobes and insula according to voxelwise analysis. Based on VOI analysis, the HPVR was positively associated with regional synaptic density in the lateral parietal (r = 0.499, *p* = 0.025), lateral temporal (r = 0.534, *p* = 0.015), and medial temporal lobes (r = 0.706, *p* = 0.001) (Figure S6B and S7B). We obtained similar results after PVC (Table S3 and Figure S3).

The MMSE score was positively associated with mGluR5 availability and synaptic density, mainly in the bilateral lateral parietal and temporal lobes according to voxelwise analysis. Based on VOI analysis, the MMSE score was positively associated with the [^18^F]PSS232 SUVr of the global (r = 0.460, *p* = 0.041) and medial temporal lobes (r = 0.842, *p* < 0.001) and with the [^18^F]SynVesT‐1 SUVr of the global (r = 0.454, *p* = 0.044) and medial temporal lobes (r = 0.697, *p* = 0.001), as shown in Figure S6C and SD. Similar results were obtained after PVC (Table S3 and Figure S3).

### 3.7 mGluR5 availability and synaptic density mediate the relationship between Aβ plaque load and global cognition

**FIGURE 6:**
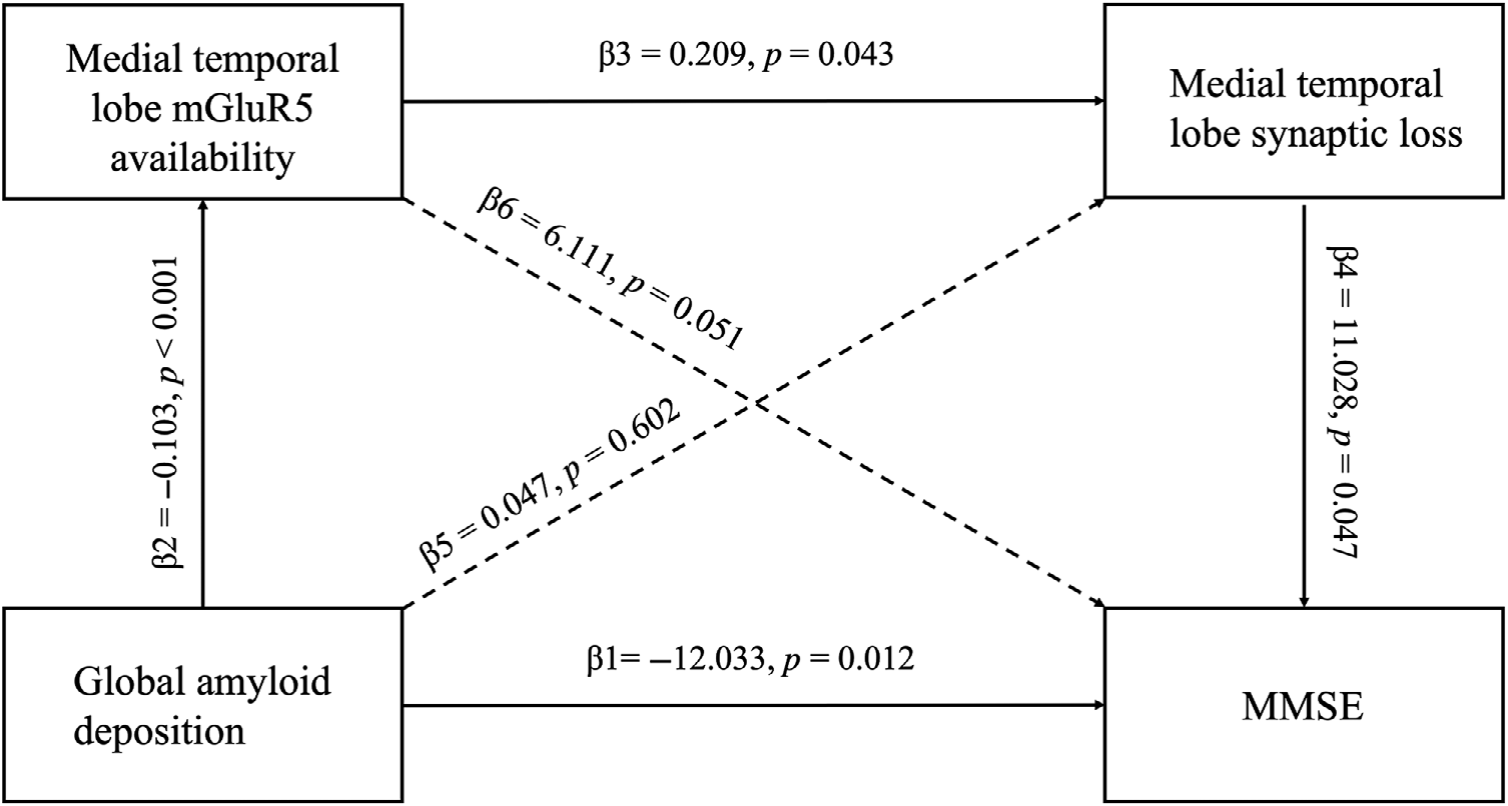
mGluR5 and synaptic density partially mediate the relationship between Aβ pathology and global cognition. Solid and dashed lines indicate significant and non‐significant pathways, respectively.

We conducted mediation analyses to further investigate the role of mGluR5 in the hallmarks of AD: Aβ deposition and synaptic loss. mGluR5 availability in the medial temporal lobe mediated the association between global amyloid deposition and synaptic density in the medial temporal lobe (β = −2.957, 95% CI: −9.861 to −0.290). mGluR5 availability and synaptic density in the medial temporal lobe mediated the association between global amyloid deposition and the MMSE score (β = −4.248, 95% CI: −10.351 to −0.464), as shown in Figure 6. Similar results were obtained after PVC. These findings indicate that aberrant mGluR5 signaling secondary to Aβ accumulation might contribute to synaptic loss, ultimately leading to cognitive decline.

## 4 DISCUSSION

mGluR5 has been hypothesized to mediate Aβ toxicity and to be linked with Aβ and tau pathology in AD, ultimately leading to synaptic dysfunction and loss.^19^ However, the associations of postsynaptic mGluR5 availability with (pre)synaptic density are unknown. Our study found that [^18^F]PSS232 and [^18^F]SynVesT‐1 PET could be used to quantify the changes in mGluR5 availability and synaptic density in the early stages of AD. mGluR5 availability and synaptic density were reduced in the medial temporal lobe in CI individuals compared with HCs. In the whole cohort, mGluR5 availability and synaptic density exhibited similar trends. Regional mGluR5 availability was positively associated with the synaptic density in the corresponding region. mGluR5 availability and synaptic density in the medial temporal lobe were negatively associated with global amyloid deposition and positively associated with HPVR and global cognition. Lower mGluR5 availability was a mediator of amyloid deposition‐induced synaptic loss, leading to further cognitive decline. In light of the strong correlation between mGluR5 availability and synaptic density, selectively modulating the availability of mGluR5 could serve as a therapeutic strategy for protecting synapses from damage in AD.

mGluR5 has been hypothesized to mediate Aβ synaptic toxicity by several mechanisms, including promoting the clustering of Aβ as an extracellular scaffold for mGluR5^19^ and serving as a coreceptor for Aβ oligomer (Aβo) bound to cellular prion protein (PrPC) for postsynaptic activation of the tyrosine kinase Fyn.^45^, ^46^ Aβo limits the lateral diffusion of mGluR5 and promotes its clustering at synapses, resulting in increased Ca^2+^ release from intracellular stores and neuronal hyperexcitability, ultimately causing synaptic deterioration.^19^, ^47^ The Aβo–mGluR5–PrPc complex elicits tau hyperphosphorylation by activating the tyrosine kinase Fyn.^46^ Pathogenic tau binds to synaptic vesicles via its N‐terminal domain and interferes with presynaptic functions, including synaptic vesicle mobility and release rate, lowering neurotransmission.^48^ Soluble forms of hyperphosphorylated tau can interact with PrPC in a manner similar to Aβ_42_ and trigger pathological signaling and synaptic dysfunction, possibly in a mGluR5‐dependent manner.^49^ Tauopathy in AD can be initiated by Aβ_42_‐mediated activation of mGluR5, and both pathological tau and Aβ_42_ oligomers act together to impair neuronal circuits and disrupt synaptic transmission.^50^ If Aβo‐induced synaptic loss occurs preferentially at mGluR5‐containing sites, then this might also account for the reduction in mGluR5 levels at synapses in the present study; mGluR5 availability and synaptic density had the same spatial pattern of alterations.

mGluR5 is mainly located in excitatory synapses and glial cells and is distributed throughout the cortex and hippocampus, where it modulates synaptic transmission.^21^ In our study, [^18^F]PSS232 PET and [^18^F]SynVesT‐1 PET revealed reduced mGluR5 availability and synaptic density in the medial temporal lobe, which is in line with previous studies.^12^, ^21^, ^22^, ^51^ Regional mGluR5 availability was positively associated with the synaptic density in the corresponding region. Synaptic density in the entire cortex and medial temporal lobe was positively associated with global and regional mGluR5 availability, and mGluR5 availability in the entire cortex and medial temporal lobe was also positively associated with global and regional synaptic density. Reductions in both mGluR5 availability and synaptic density were observed in the medial temporal lobe in CI individuals. Moreover, mGluR5 availability was closely associated with synaptic density. Aberrant mGluR5 levels may influence the regional pattern of synaptic loss, contributing to AD pathogenesis.^21^

Our study found that composite cortical amyloid deposition was negatively associated with mGluR5 availability and synaptic density in the medial temporal lobe. *Post mortem* studies on brain tissue slices revealed lower mGluR5 availability and synaptic density in the hippocampus of AD patients compared to those of HCs. This is consistent with previous studies, which showed that global cortex amyloid deposition was negatively associated with SV2A binding in the medial temporal lobe^51^ and that this association was dependent on mGluR5 availability in both the entire cortex and medial temporal lobe. We also found that mGluR5 availability in the entire cortex/medial temporal lobe mediated the relationship between global amyloid deposition and synaptic density in the medial temporal lobe. This may indicate that reductions in mGluR5 availability secondary to Aβ aggregation might promote synaptic dysfunction and loss. Synaptic loss in a mouse model of AD can be reversed by targeting mGluR5.^24^ These findings may indicate that aberrant mGluR5 signaling has a deleterious effect on synapses and that Aβo can lead to synaptic loss through binding to mGluR5.^49^

We also found that hippocampal volume was positively associated with mGluR5 availability and synaptic density in most brain regions. The medial temporal lobe, especially the hippocampus, is one of the brain regions affected earliest by AD‐related pathological changes, such as atrophy.^52^ In AD, atrophy of the medial temporal lobe is a biomarker similar to glucose hypometabolism. A previous study showed a concordant reduction in synaptic density and metabolism in the medial temporal lobe in the same AD patients.^53^ Aberrant mGluR5 signaling has been shown to play a central role in regulating both Aβ42 and tau pathological signaling and triggering synaptic dysfunction.^49^ Whether mGluR5 availability is linked to neurodegeneration during the progression of AD needs to be further studied.

mGluR5, which is ubiquitously expressed in the brain, is considered to be partially responsible for memory and learning and plays a key role in regulating neuronal transmission and synaptic strength, and it has been suggested that aberrant mGluR5 signaling is involved in mediating multiple aspects of cognitive deficits associated with AD.^47^, ^54^, ^55^ A previous study showed that hippocampal mGluR5 binding was associated with episodic memory scores and global function.^21^ Our study found that mGluR5 binding in the global and medial temporal lobe was positively associated with the MMSE score. We also found that synaptic density in the global and medial temporal lobes was positively associated with the MMSE scores, which is similar to the results reported by Mecca et al.^5^ mGluR5 availability and synaptic density in the medial temporal lobe were found to mediate the association between global amyloid deposition and the MMSE score. These findings indicate that aberrant mGluR5 signaling secondary to Aβ accumulation might contribute to synaptic loss, ultimately leading to cognitive decline. Taken together, these findings indicate that mGluR5 availability and synaptic density are closely related to global cognition. These findings have important clinical implications, providing possible targets for therapies to improve cognition in AD patients.

This study has several limitations. First, the sample size was relatively small. For this reason, we need to be careful when drawing far‐reaching conclusions from these preliminary findings. Second, we did not further divide the participants into females and males to assess the effect of sex on mGluR5 expression, as previous studies reported that sex could affect mGluR5 expression in the brains of AD patients.^1^ Third, we included only patients with MCI and early AD; there were no patients with moderate to severe AD. Further, we only included Aβ‐negative HCs and did not investigate a continuum between the accumulation of amyloid and reductions in mGluR5 availability and synaptic density. Subsequent studies will include subjects at different stages for analysis. Finally, our study was cross‐sectional and did not involve systematic and comprehensive observation of longitudinal changes in mGluR5 expression and synaptic density.

In summary, decreases in mGluR5 availability and synaptic density follow a similar spatial pattern in the brains of CI patients. mGluR5 availability was closely associated with synaptic density throughout the cortex and in the medial temporal lobe. mGluR5 availability and synaptic density were both negatively associated with amyloid deposition and positively associated with hippocampal volume and global cognition. mGluR5 availability and synaptic density mediate the relationship between amyloid deposition and global cognition, suggesting that lower mGluR5 availability secondary to Aβ deposition might accelerate synaptic loss, leading to further cognitive decline. Modulating mGluR5 availability might be a potential therapeutic strategy for improving synaptic function in AD.

## CONFLICT OF INTEREST STATEMENT

The authors report that they have no conflicts or competing interests. Author disclosures are available in the supporting information.

## CONSENT STATEMENT

This manuscript was seen and approved by all authors. All authors declare no conflicts of interest that might directly or indirectly influence the content of the manuscript submitted.

## Supporting information

Supporting Information

Supporting Information
