# Supplementary material for: Presynaptic density determined by SV2A PET is closely associated with postsynaptic metabotropic glutamate receptor 5 availability and independent of amyloid pathology in early cognitive impairment
Source: Alzheimers Dement. 2024 Apr 18;20(6):3876–88. doi: 10.1002/alz.13817 (PMC11180932; doi:10.1002/alz.13817)
Supplement: Supplementary file 1 — Supporting Information [file ALZ-20-3876-s001.docx]

**Presynaptic density determined by SV2A PET is closely associated with postsynaptic metabotropic glutamate receptor 5 availability and independent of amyloid pathology in early cognitive impairment**

**Supplemental Table 1. Detailed information on post-mortem human brain tissues**

| **Group** | **n** | **Age (y)** | **Sex** | **APOE** **ε4** **(0/1/2)** | **PMD (h)** | **Braak** | **Amyloid-β** |
| --- | --- | --- | --- | --- | --- | --- | --- |
| AD | 5 | 72 ± 8.7 | 4F/1M | 2/2/1 | 4:54 ± 0:29 | 4-6 | C |
| HC | 5 | 82 ± s2.5 | 2F/3M | 4/1/0 | 5:58 ± 0:27 | 0-3 | O-C |

Abbreviations: F: female; M: male; APOE: apolipoprotein E; PMD: Postmortem delay.

**Supplemental Table 2. The information on the primary and 2^nd^ antibodies in detail**

| Reagent/Antibodies | Source | Identifier | Dilution |
| --- | --- | --- | --- |
| Rabbit anti-mGluR5 | Abcam | ab76316 | 1:500 |
| Mouse anti-Abeta (6E10, IF) | Biolegend | 803004 | 1:1000 |
| Rabbit anti-SV2A | Abcam | ab32942 | 1:500 |
| AlphaTSA Multiplex IHC Kit | AlphaX Biotech | AXT37100031 | 1:100 |
| XTSA570 | AlphaX Biotech | AXT6410000 | 1:100 |
| XTSA480 | AlphaX Biotech | AXT6110000 | 1:100 |
| XTSA670 | AlphaX Biotech | AXT9910000 | 1:100 |
| Mouse purified anti-β-Amyloid, 17-24 monoclonal antibody (4G8, IHC) | 800701 | Biolegend | 1:4000 |
| Antibody diluent/block | AlphaX Biotech | AXT9310000 | / |
| Ethylenediaminetetraacetic acid | Zsbio | ZLI-9079 | 1:50 |
| Mounting medium | Phygene | PH0429 | / |
| DAPI | Invitrogen | P36970 | 1:10 |

Abbreviations: DAPI: 4’, 6-diamidino-2-phenylindole; SV2A: Synaptic vesicle glycoprotein 2A.

**Supplemental Table 3. The associations among mGluR5 availability, synaptic density, global amyloid deposition, HPVR and MMSE based on ROI analysis with PVC in the CI + HCs**

|  | global [^18^F] SynVesT-1 SUVr | Frontal [^18^F]  SynVesT-1 SUVr | LP [^18^F]  SynVesT-1 SUVr | LT [^18^F]  SynVesT-1 SUVr | MT [^18^F]  SynVesT-1 SUVr | PC [^18^F]  SynVesT-1 SUVr | Precuneus [^18^F] SynVesT-1 SUVr | Occipital [^18^F]  SynVesT-1 SUVr | Global [^18^F] Florbetapir  SUVr | MMSE |
| --- | --- | --- | --- | --- | --- | --- | --- | --- | --- | --- |
|  | r, p value | r, p value | r, p value | r, p value | r, p value | r, p value | r, p value | r, p value | r, p value | r, p value |
| global [^18^F]  PSS232 SUVr | 0.169, 0.478 | 0.023, 0.923 | 0.115, 0.630 | **0.305, 0.048** | **0.442, 0.040** | 0.012, 0.960 | 0.187, 0.126 | 0.150, 0.327 | -0.378, 0.100 | -0.288, 0.218 |
| MT [^18^F]  PSS232 SUVr | **0.492, 0.028** | -0.235, 0.320 | **0.565, 0.009** | 0.382, 0.097 | **0.734, < 0.001** | 0.009, 0.971 | **0.563, 0.010** | **0.415, 0.069** | **-0.773, <0.001** | **0.711, <0.001** |
| HPVR | **0.503, 0.024** | -0.366, 0.112 | **0.540, 0.014** | **0.649, 0.002** | **0.569, 0.009** | 0.377, 0.102 | **0.478, 0.033** | **0.542, 0.014** | N/A | N/A |
|  | global [^18^F] PSS232 SUVr | Frontal [^18^F]  PSS232 SUVr | LP [^18^F] PSS232 SUVr | LT [^18^F] PSS232 SUVr | MT [^18^F] PSS232 SUVr | PC [^18^F] PSS232 SUVr | Precuneus [^18^F] PSS232 SUVr | Occipital [^18^F]  PSS232 SUVr | Global [^18^F] Florbetapir SUVr | MMSE |
|  | r, p value | r, p value | r, p value | r, p value | r, p value | r, p value | r, p value | r, p value | r, p value | r, p value |
| global [^18^F] SynVesT-1 SUVr | 0.169, 0.478 | **0.581, 0.007** | **0.491, 0.028** | **0.542, 0.014** | **0.492, 0.028** | **0.484, 0.031** | **0.454, 0.044** | **0.467, 0.038** | -0.220, 0.351 | **0.500, 0.025** |
| MT [^18^F]  SynVesT-1 SUVr | -0.122, 0.609 | **0.608, 0.004** | **0.565, 0.009** | **0.675, 0.001** | **0.734, <0.001** | **0.556, 0.017** | **0.689, 0.001** | **0.594, 0.006** | **-0.489, 0.029** | **0.689, 0.001** |
| HPVR | -0.329, 0.156 | 0.246, 0.296 | 0.195, 0.411 | 0.432, 0.057 | **0.484, 0.031** | 0.360, 0.119 | 0.125, 0.599 | 0.375, 0.104 | N/A | N/A |

The statistical model is Pearson’s correlation, with age, education years, and gender as covariates.

Abbreviations: LP: lateral parietal lobe; LT: lateral temporal lobe; MT: medial temporal lobe; PC: posterior cingulate; SUVr: standardized uptake value ratio; HPVR: hippocampal volume ratio. NOTE: Bolded text indicates statistical significance (p < 0.05).


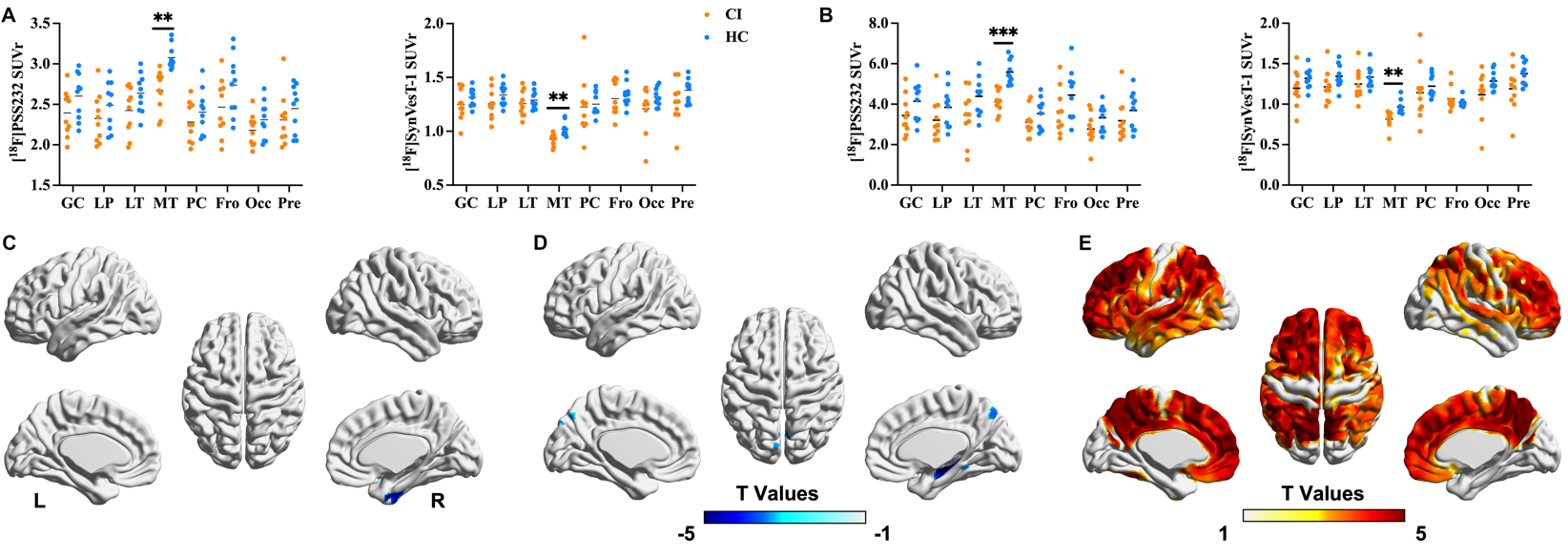


**Supplemental Figure 1. Group differences in mGluR5 availability, synaptic density and amyloid deposition between CI and HC groups.**  (A-B) Group differences in mGluR5 availability and synaptic density between CI and HC groups without and with partial volume corrected results, based on VOI-analysis. (C-E) Group differences in mGluR5 availability, synaptic density and amyloid deposition between CI and HC groups with partial volume corrected results, based on voxelwise-analysis.

All p-values less than 0.05 are marked in the figure. *, ** and *** indicate significant differences between the two groups with p < 0.05, p < 0.01 and p < 0.001, respectively. The colors indicate the T value of the differences between groups with a statistical threshold of p < 0.001 and a minimum cluster extent ke of ≥ 100 voxels according to voxelwise analysis. The analyses were adjusted for age, gender, and years of education as covariates.

Abbreviations: GC: global cortex; LP: lateral parietal lobe; LT: lateral temporal lobe; MT: medial temporal lobe; PC: posterior cingulate; Fr: frontal lobe; Occ: Occipital lobe; Pre: Precuneus; SUVr: standardized uptake value ratio.

**
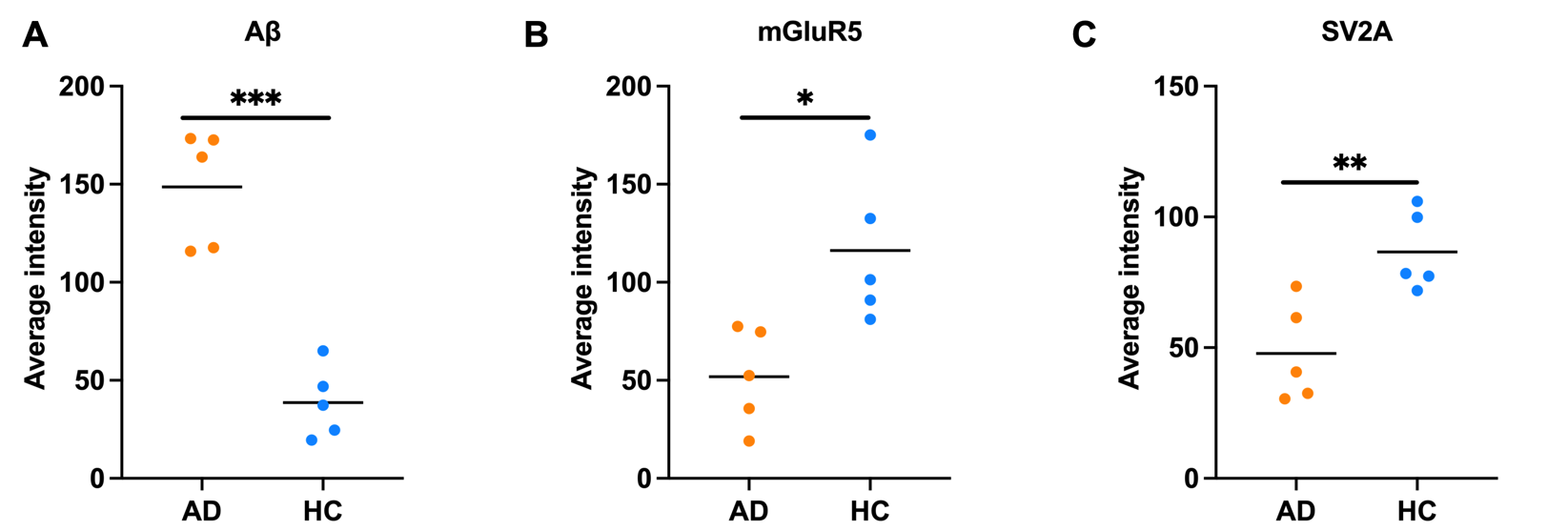
**

**Supplemental Figure 2. Quantitative analysis of immunofluorescence staining of Aβ, mGluR5 and SV2A in the postmortem hippocampus slices of AD and HCs.** The average fluorescence intensity of Aβ was higher in the hippocampus from AD patients (n=5) than in HCs (n=5), while the average fluorescence intensity of mGluR5 and SV2A was lower in the hippocampus from AD patients (n=5) than HCs (n=5). *, ** and *** indicate significant differences between the two groups with p < 0.05, p < 0.01 and p < 0.001, respectively.


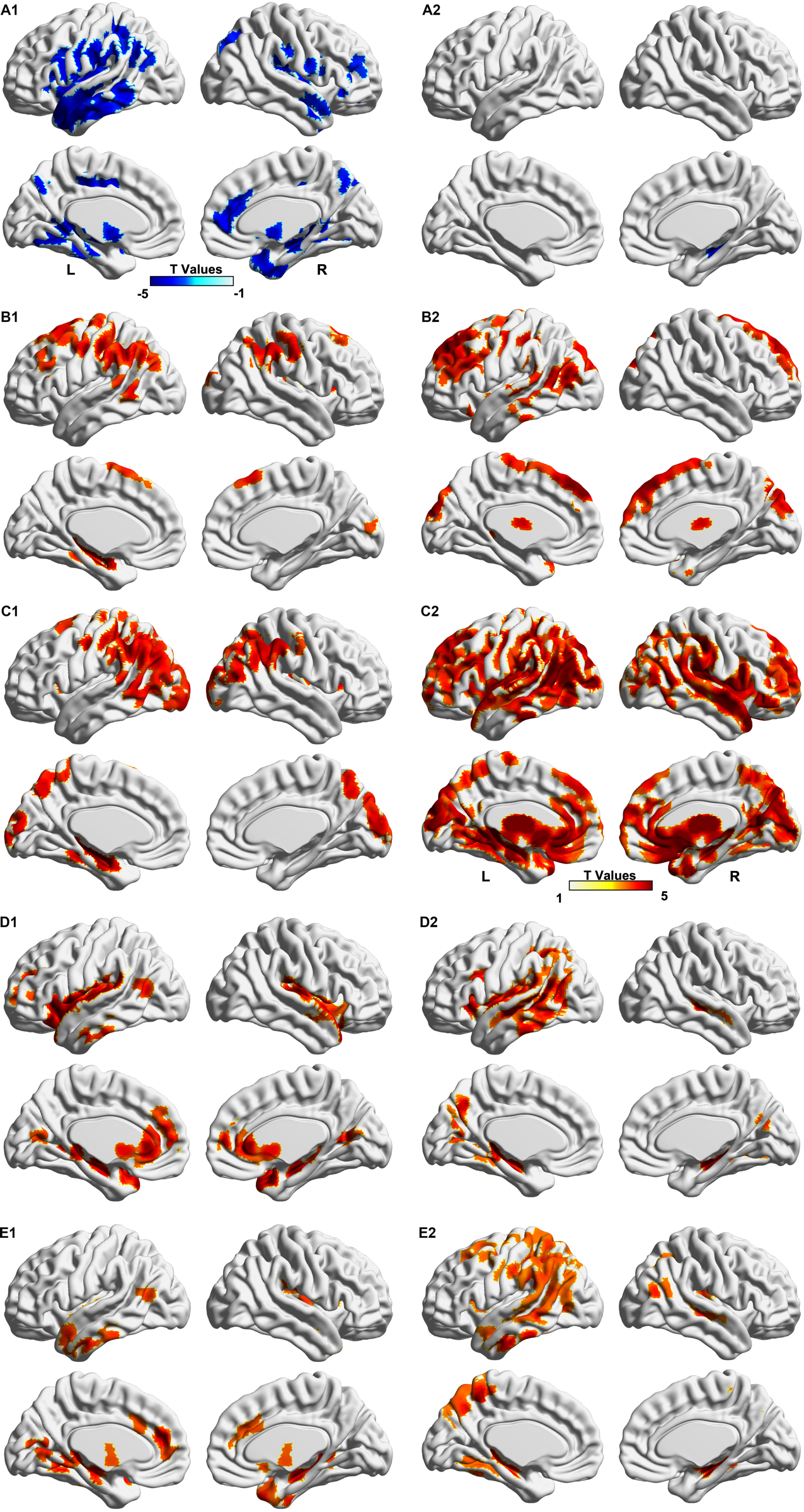


**Supplemental Figure 3. The associations of global amyloid deposition, hippocampal volume and cognition with synaptic density and mGluR5 availability.**

A. the associations of global amyloid deposition with mGluR5 availability(A1) and synaptic density (A2);

B1. the associations of global mGluR5 availability with synaptic density; B2. the associations of global synaptic density with mGluR5 availability;

C1. the associations of mGluR5 availability in the medial temporal lobe with synaptic density; C2. the associations of synaptic density in the medial temporal lobe with mGluR5 availability;

D. the associations of hippocampal volume with mGluR5 availability (D1) and synaptic density (D2);

E. the associations of MMSE scores with mGluR5 availability (E1) and synaptic density (E2).

The colors indicate the T value of the differences between groups with a statistical threshold of p < 0.001 and a minimum cluster extent ke of ≥ 100 voxels according to voxelwise analysis. The analyses were adjusted for age, gender, and years of education as covariates.


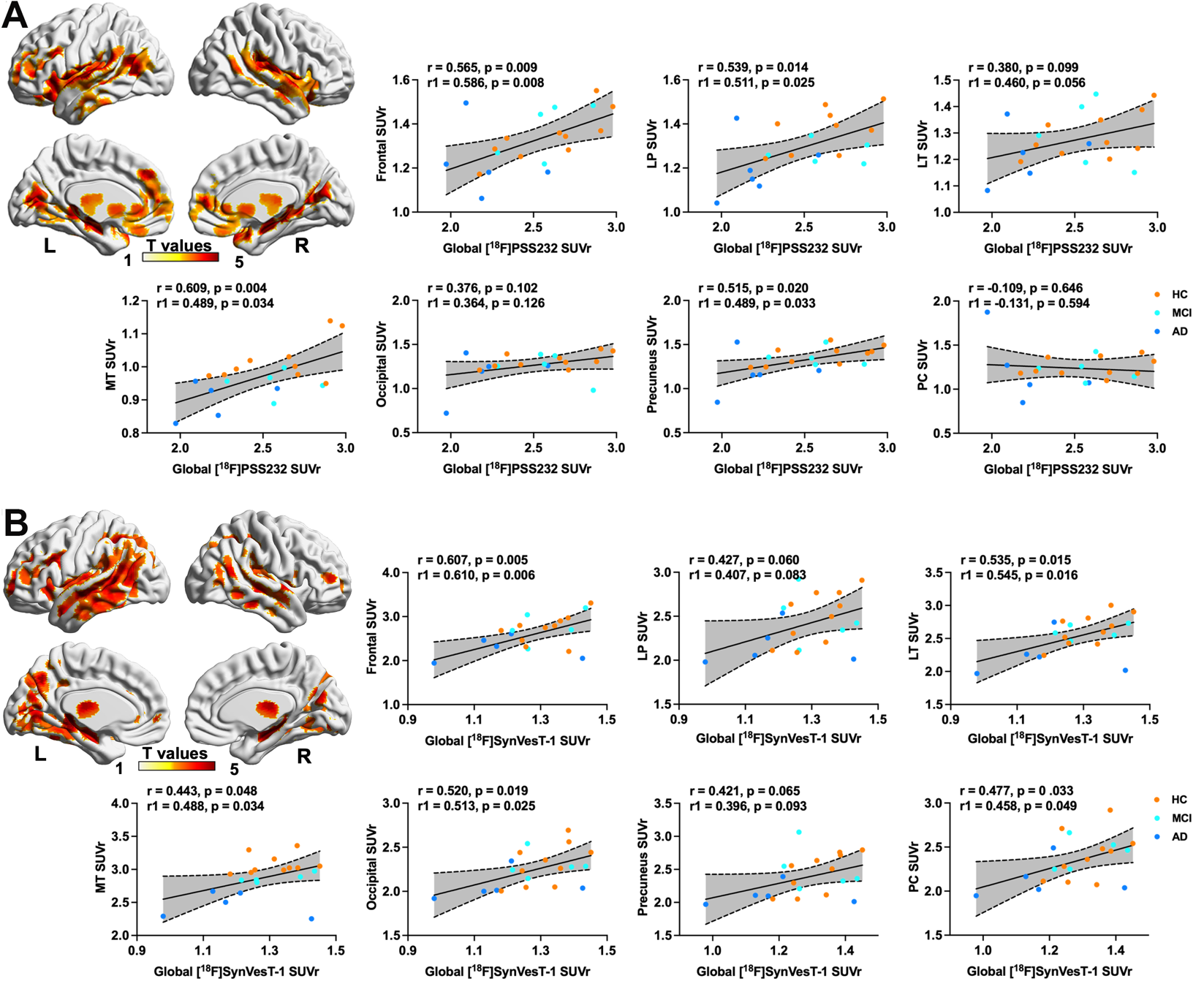


**Supplemental Figure 4. The associations between mGluR5 availability and synaptic density in the whole cohort.**

(A). The association between global mGluR5 availability and synaptic density; (B). The association between global synaptic density and mGluR5 availability. The colors represent the T value with a statistical threshold of p < 0.001 and a minimum cluster extent ke of ≥ 100 voxels. The statistical model is Pearson’s correlation and the dashed lines represent the 95% confidence intervals of the best-fit lines. r: with age, education years, and gender as covariates; r_1_: with addition global amyloid deposition as a covariate.

Abbreviations: GC: global cortex; LP: lateral parietal lobe; LT: lateral temporal lobe; MT: medial temporal lobe; PC: posterior cingulate; Fr: frontal lobe; Occ: Occipital lobe; Pre: Precuneus; SUVr: standardized uptake value ratio.


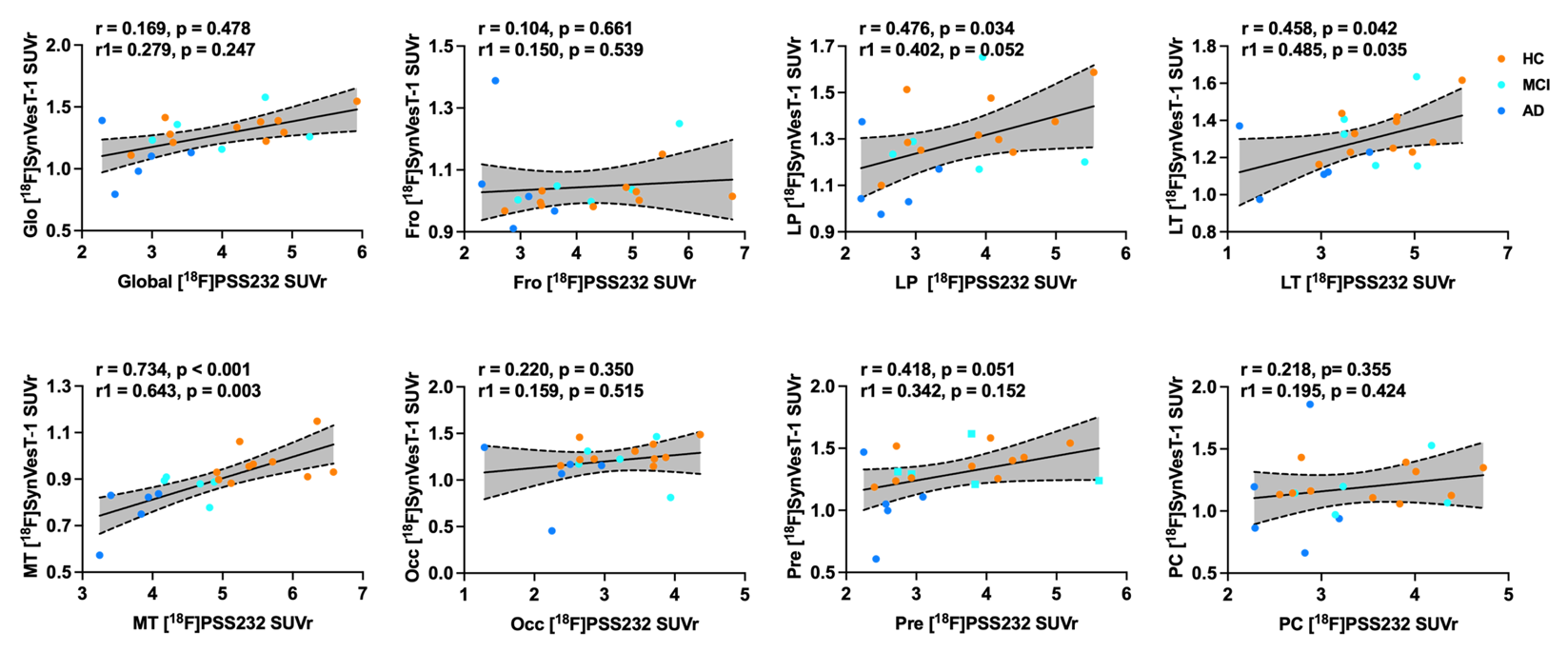


**Supplemental Figure 5. The associations between regional mGluR5 availability and regional synaptic density,** using PVC PET images analyzed. Pearson’s correlation analysis was performed; the dashed lines represent the 95% confidence intervals of the best-fit lines. r: with age, education years, and gender as covariates; r_1_: with addition global amyloid deposition as a covariate. Abbreviations: GC: global cortex; LP: lateral parietal lobe; LT: lateral temporal lobe; MT: medial temporal lobe; PC: posterior cingulate; Fr: frontal lobe; Occ: Occipital lobe; Pre: Precuneus; SUVr: standardized uptake value ratio.


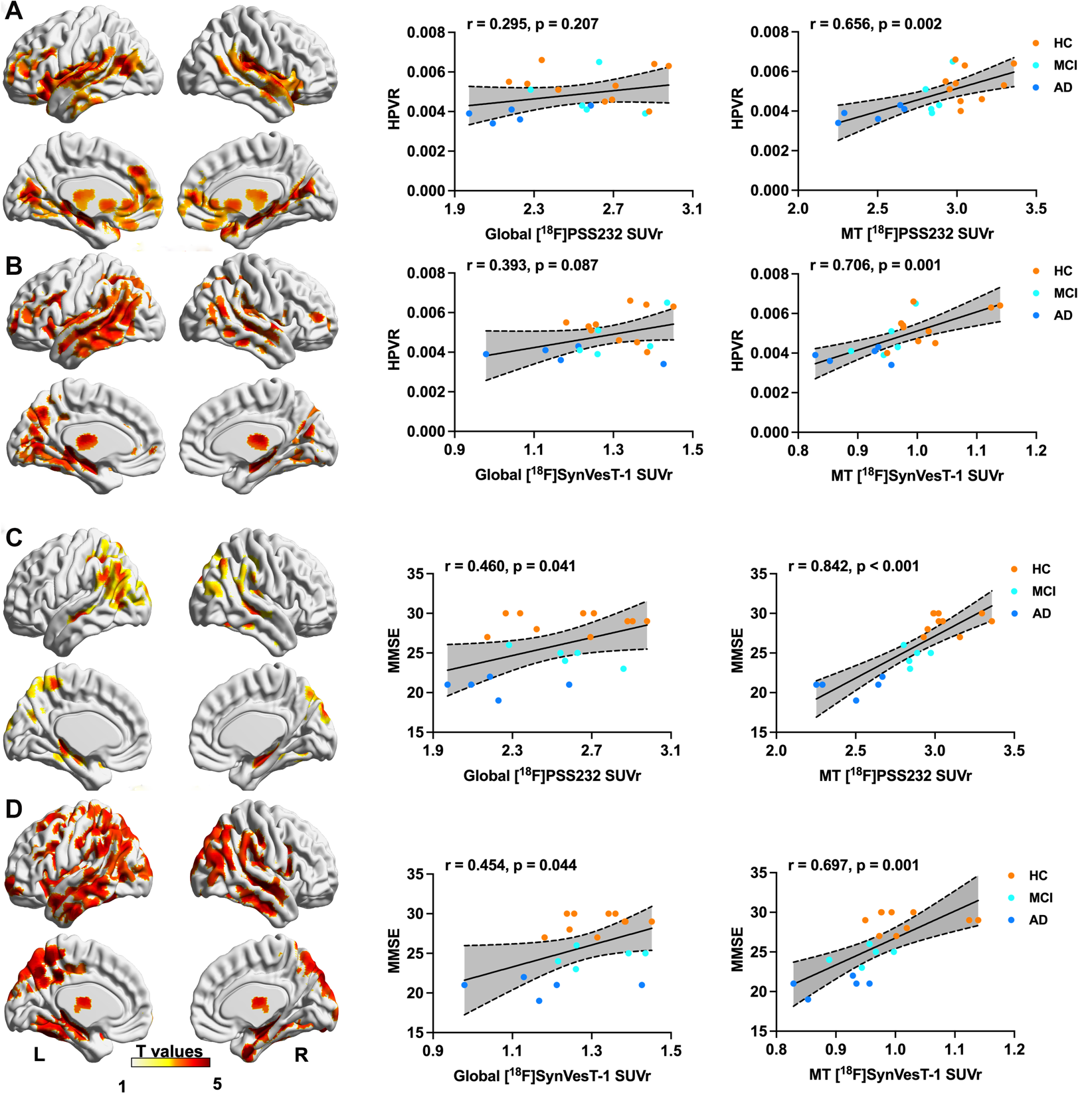


**Supplemental Figure 6. The associations of HPVR/MMSE** **score with mGluR5 availability and synaptic density in the whole cohort.**

A. The associations of HPVR with mGluR5 availability; B. The associations of HPVR with synaptic density;

C. The associations of MMSE score with mGluR5 availability; D. The associations of MMSE score with synaptic density.

The colors represent the T value with a statistical threshold of p < 0.001 and a minimum cluster extent ke of ≥ 100 voxels. Pearson’s correlation analysis was performed; the dashed lines represent the 95% confidence intervals of the best-fit lines.

Abbreviations: MT: medial temporal lobe; HPVR: hippocampal volume ratio.

**
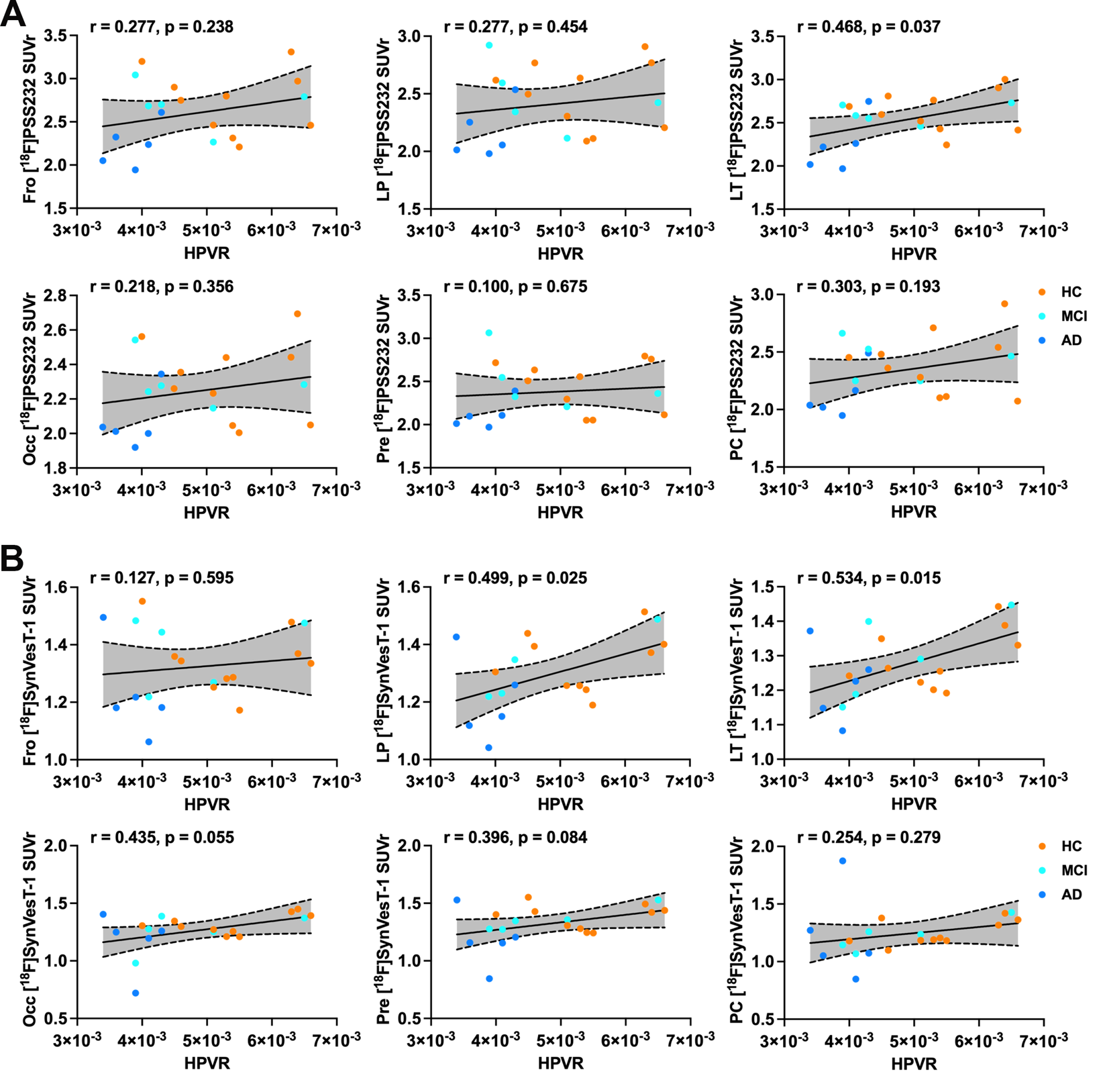
**

**Supplemental Figure 7. The associations of HPVR with regional (A) mGluR5 availability and (B) synaptic density in the whole cohort.**

(A). The associations of HPVR with regional mGluR5 availability; (B). The associations of HPVR with regional synaptic density. Abbreviations: HPVR: hippocampal volume ratio; LP: lateral parietal lobe; LT: lateral temporal lobe; MT: medial temporal lobe; PC: posterior cingulate; Fro: frontal lobe; SUVr: standardized uptake value ratio.
